# First Carpo-metacarpal Joint Arthritis: Interpositional Arthroplasty using Trapezium

**DOI:** 10.7759/cureus.861

**Published:** 2016-11-03

**Authors:** Chirag Kapoor, Ankur Kansagra, Maulik Jhaveri, Aditya Merh, Paresh Golwala

**Affiliations:** 1 Orthopaedics, Sumandeep Vidyapeeth, Vadodara, Gujarat

**Keywords:** carpo-metacarpal, arthritis, trapezium

## Abstract

Thumb pain secondary to arthritis at the basal joint of the thumb is a common condition, especially in women, and can be quite disabling. An accurate diagnosis can be readily made from the history and examination. Reconstructive procedures for each stage of the disease are aimed at relieving pain and restoring thumb motion and strength. There are a number of methods available to treat this condition both conservatively and surgically with variable success rates. We present a case of a middle-aged female with first carpo-metacarpal (CMC) joint arthritis in whom we have tried a new technique in which the trapezium is excised, crushed, put in a sponge covering and then inserted back in the void created after excision. At the one-year follow-up, the patient was pain-free and had full range of thumb movement.

## Introduction

Carpo-metacarpal (CMC) joint arthritis is a prevalent condition affecting up to 10% of middle-aged women [1]. The basal joint of the thumb consists of four trapezial articulations: the trapeziometacarpal (TM), trapeziotrapezoid, scaphotrapezial (ST), and trapezium-index metacarpal articulations. Only the TM and ST joints lie along the longitudinal compression axis of the thumb. North and Eaton have observed that radiographic disease most commonly affects these two joints [2]. There are many treatment options available with variable results.

Conservative treatment is considered in early stages of the disease while advanced stages have to be dealt with surgically. We present a case of a middle-aged female with symptomatic late stage first CMC joint arthritis who was treated surgically by a new technique, with an excellent outcome. Informed consent was obtained from the patient for this study.

## Case presentation

The patient was a 55-year-old female with a six-month history of pain in the base of the right thumb. The pain was insidious at onset, gradually progressing, non-radiating, and it increased on activity. She had a weak grip and had pain when she made a pinching movement. She had no other comorbidities or associated conditions like diabetes or rheumatoid arthritis.

On examination, there was tenderness and swelling at the base of the right thumb without any signs of inflammation. The thumb CMC grind test was positive. The thumb active range of movement was restricted in flexion, circumduction, and opposition as compared to the normal thumb. Further passive movement was possible but was painful. Metacarpo-phalangeal (MCP) joint hyperextension instability was present as compared to the contralateral side. There was no associated neurovascular deficit with normal sensations and motor power of the thumb.

**Figure 1:**
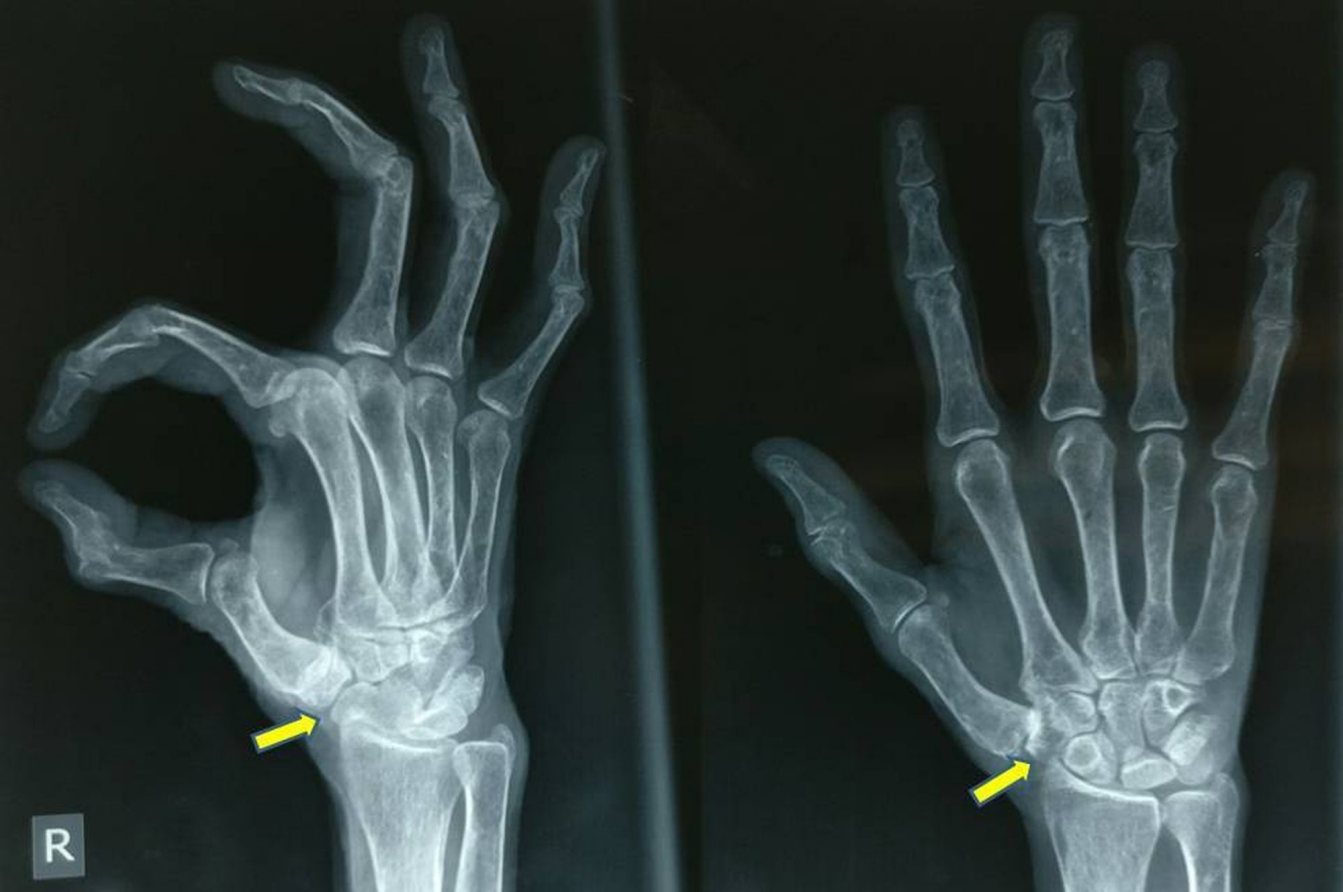
Pre-operative radiograph (antero-posterior and oblique views) Image showing arthritic first metacarpal-trapezium joint.

Plain radiographs revealed degeneration of the first trapeziometacarpal joint, i.e. first CMC joint, with irregular and sclerosed joint margins and a few osteophytes on the radial aspect of the thumb base (Figure 1).

**Figure 2:**
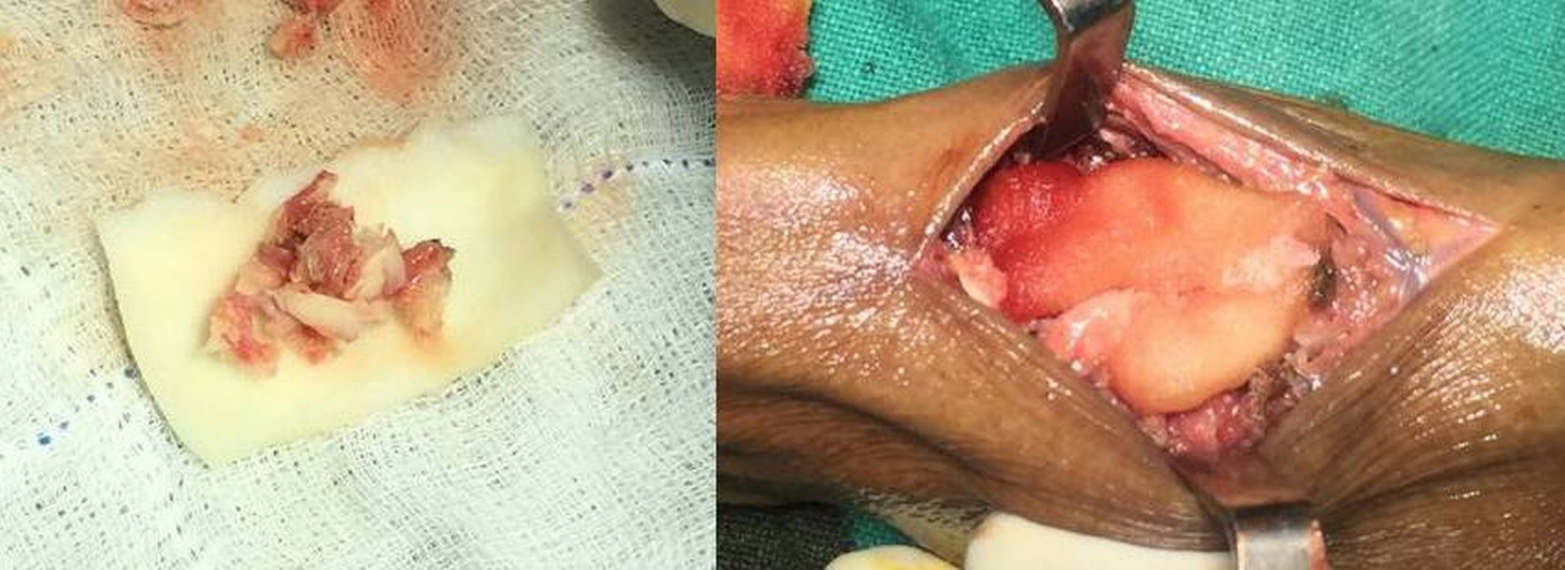
Operative technique Left: trapezium crushed and put inside a sponge covering. Right: trapezium graft put inside the cavity created after trapeziectomy.

After obtaining a written informed consent from the patient, she was submitted to surgery. A dorsal longitudinal incision was taken centring over the CMC joint till the thenar eminence, with care taken to protect the superficial branches of the radial nerve. The trapezium was exposed and excised in toto. The degenerated cartilage at the base of the first metacarpal was shaved off. The excised trapezium was crushed to small pieces and put inside a wet sponge piece which completely engulfed it (Figure 2). This sponge with the bone pieces was put in the cavity that was created after the excision of the trapezium (Figure 2).

**Figure 3:**
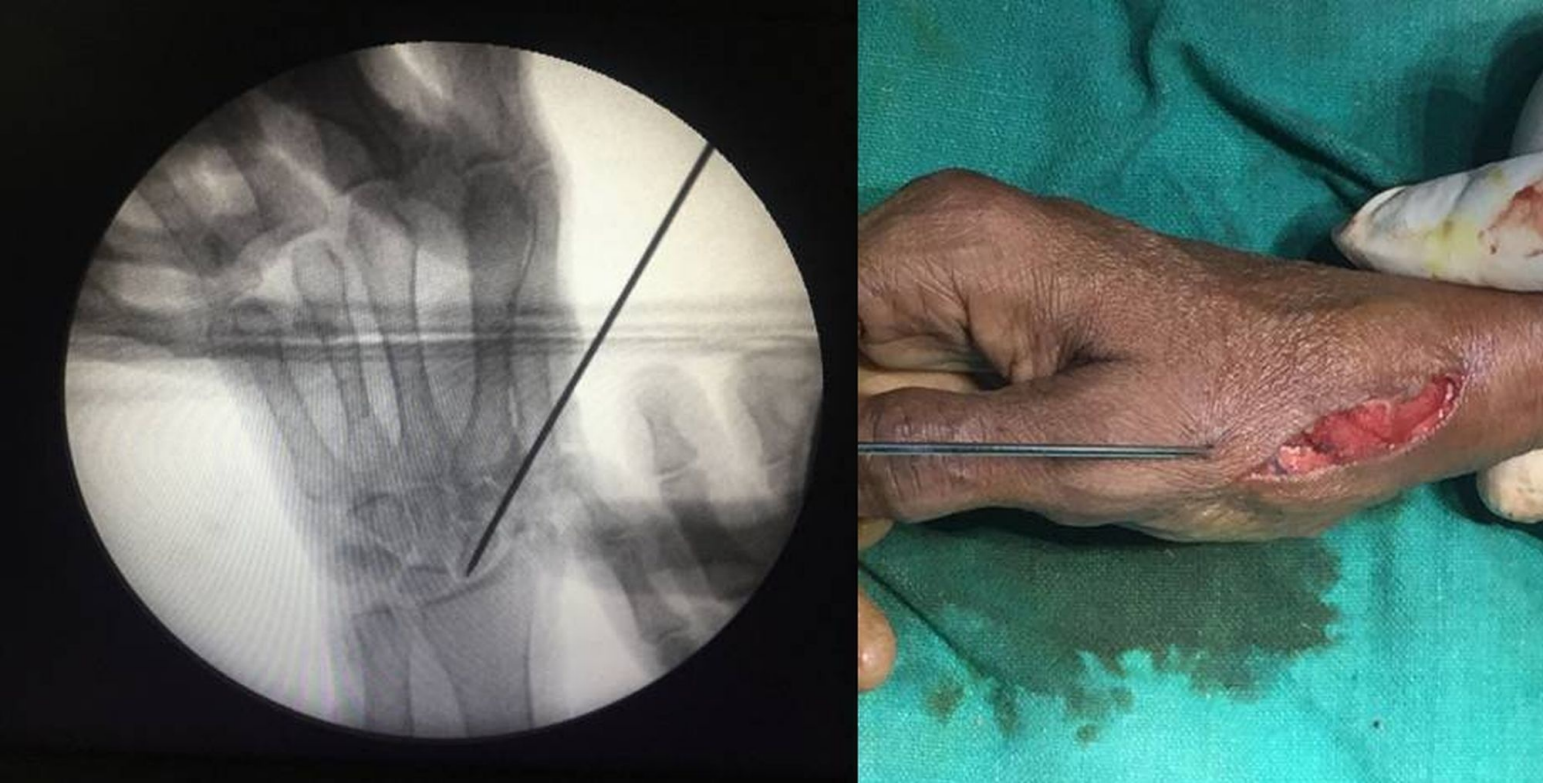
Intraoperative images Image showing K-wire transfixed in metacarpal and scaphoid.

**Figure 4:**
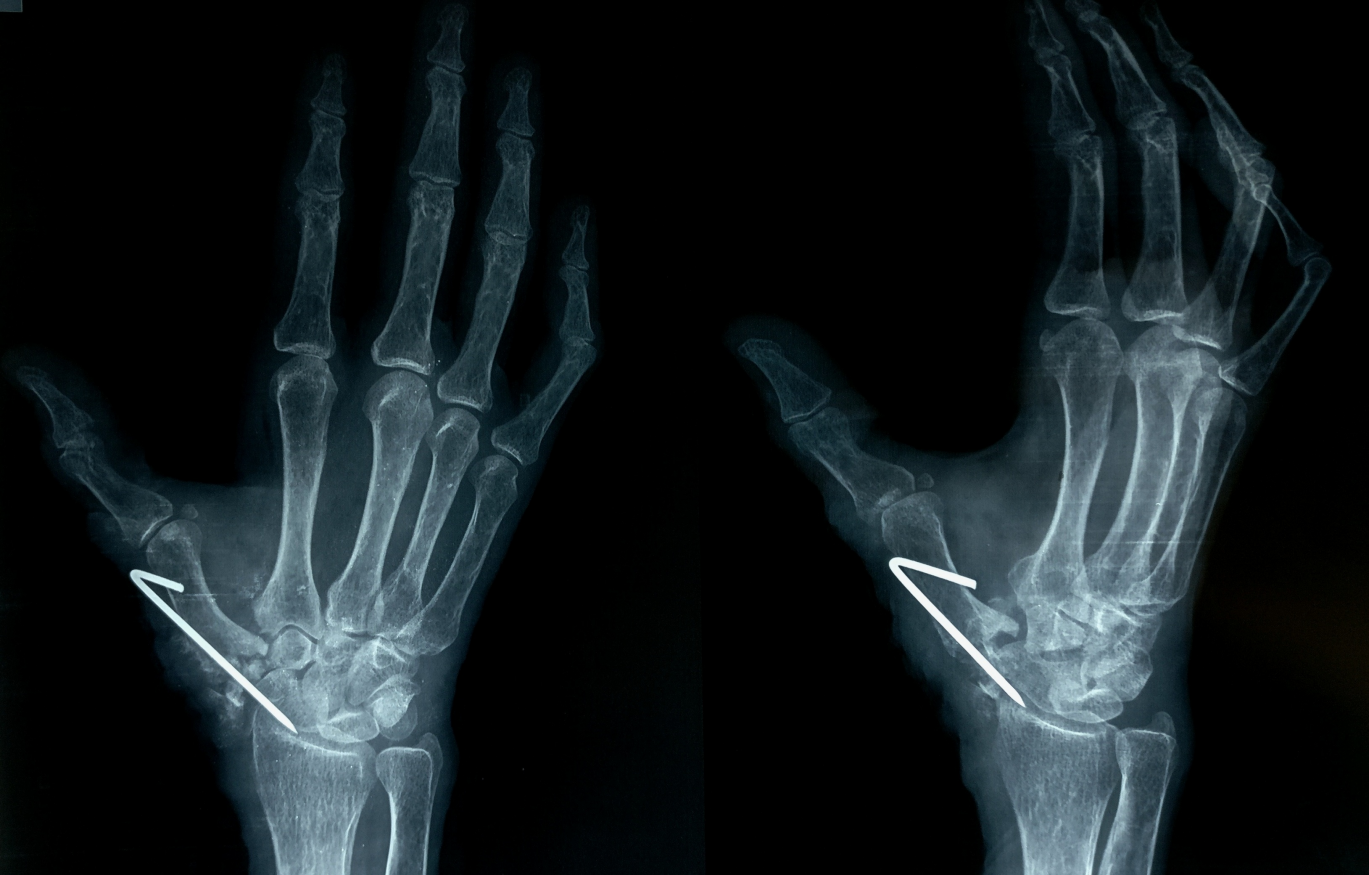
Postoperative radiographs (antero-posterior and oblique views) Image showing K-wire in situ and cavity filled up with trapezium graft.

A 2 mm K-wire was passed starting from the base of the first metacarpal through the sponge piece and transfixed into the scaphoid (Figures 3-4). This was done as a support to the joint for postoperative immobilization, along with a below-elbow scaphoid cast for six weeks. The K-wire and cast were then removed, and she was started with thumb range of movement exercises (active and passive) for two months.

**Figure 5:**
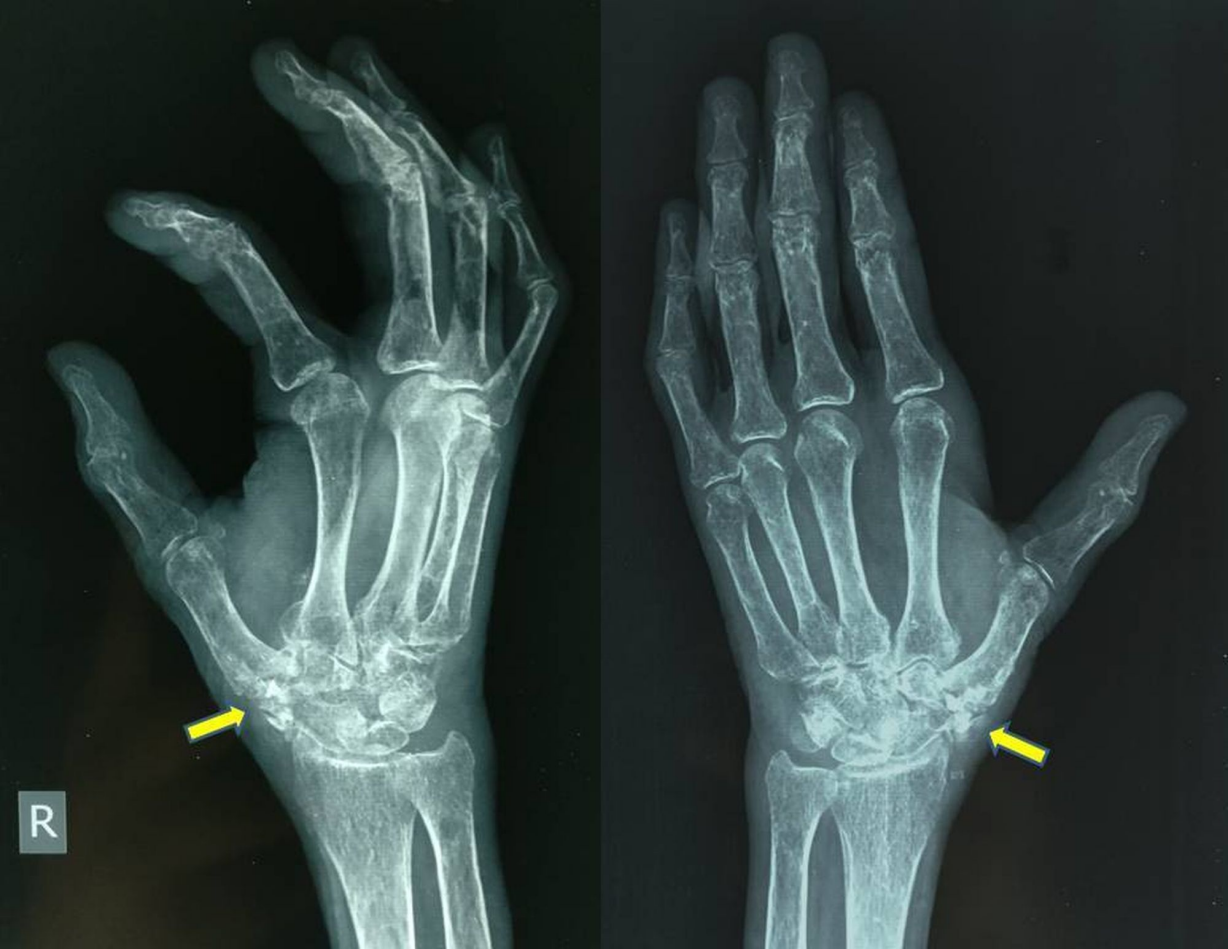
Follow-up radiograph at one year Image showing the trapezium that had gotten remodelled to create a pseudojoint.

At the one-year follow-up, the patient had no pain with full range of thumb movements. Follow-up radiographs show that the graft engulfed in the sponge was still in place and had gotten remodelled to create a pseudojoint (Figure 5).

## Discussion

Carpo-metacarpal (CMC) osteoarthritis, also known as trapezio-metacarpal osteoarthritis or osteoarthritis at the base of the thumb is a reparative joint disease affecting the first carpo-metacarpal joint [3]. This joint is formed by the trapezium bone of the wrist and the first metacarpal bone of the thumb. Because of its relative instability, this joint is a frequent site for osteoarthritis [1].

It is believed that laxity of the ligaments especially the palmar beak ligament, surrounding the first CMC joint is the main cause of arthritis [4]. This instability causes misalignment of the joint bones, which will then rub against each other, which causes wearing of the cushioning cartilage of the joint surfaces, resulting in damage of the joint [5].

It is commonly seen in obese females after menopause, as was also seen in our case [6]. Tenderness is usually well localised over the joint, and this can be reproduced with thumb and finger pressure applied directly over the affected joint. Crepitus evident on examination implies erosion of the articular cartilage [7]. All these features were consistent with our patient.

There are numerous conservative treatment options which include application of splints and slings, analgesics and injection of steroids, but these can be used in early stages of the disease only and in less symptomatic patients [2]. For advanced stage arthritis, various surgical procedures like excision of the trapezium (trapeziectomy) with ligament reconstruction, with or without tendon interposition arthroplasty have been described [8-9].

Recently, some surgeons have started doing trapezial excision alone referred to as 'haematoma arthroplasty [10] and have had favourable short term results, although loss of trapezial height with subsequent scaphoid impingement is a feared long-term consequence which may affect long-term outcome. In an attempt to prevent this collapse, several alternatives to the simple trapeziectomy have been popularized. These include interposing autogenous or alloplastic tissue between the carpals and the base of the metacarpal, reconstructing the supporting ligaments with various tendons and CMC arthrodesis. Arthrodesis has the disadvantage of limitation of joint movement, and ligament reconstruction requires technical expertise with surgical morbidities.

Our method of reconstruction includes removal of the trapezium and crushing it and then engulfing it in a sponge covering. Our hypothesis is that this will allow for a new pseudojoint to be formed between the trapezium and the base of the first metacarpal without causing it to fuse because of the sponge covering, thus retaining movement at the joint. Also, this will avoid the need of ligament reconstruction and interposition, thus reducing morbidity. Moreover, filling the gap with this graft is preferable in terms of function, stability, and position of the thumb as it avoids the complications such as shortening or subluxation of the thumb.

## Conclusions

There are many surgical methods to treat this condition, but all are associated with a few demerits. We feel that our method of treatment has no associated comorbidities and requires less technical expertise, but at the same time offers excellent results. So we feel this innovative technique is a worthwhile approach for treating CMC joint arthritis.
